# Supplementary material for: Bearing fault diagnosis method based on enhanced VMD and adaptive-optimized SDAE
Source: PLoS One. 2025 Dec 4;20(12):e0337832. doi: 10.1371/journal.pone.0337832 (PMC12677484; doi:10.1371/journal.pone.0337832)
Supplement: Attachment — Submitted filename: Response to Reviewers.docx [file pone.0337832.s001.docx]

Dear Editor and Reviewers:

Thank you for your letter and for the reviewers’ comments concerning our manuscript entitled “Bearing fault diagnosis method based on enhanced VMD and adaptive-optimized SDAE”. Those comments are valuable and very helpful for revising and improving our paper, as well as the important guiding significance to our researches. We have studied comments carefully and have made corrections which we hope meet with your approval.

In the following pages are our detailed responses to each comment and the detail revisions are in the revised manuscript. We hope that the revisions in the manuscript and our accompanying responses will be sufficient to make our manuscript suitable for publication in *PlOS ONE*.

We shall look forward to hearing from you at your earliest convenience.

Sincerely yours!

Haowen Li

University of Electronic Science and Technology of China

[202322040545@std.uestc.edu.cn](mailto:202322040545@std.uestc.edu.cn)

Comment for the Academic Editor:

1. Please ensure that your manuscript meets PLOS ONE's style requirements, including those for file naming. The PLOS ONE style templates can be found at <https://journals.plos.org/plosone/s/file?id=wjVg/PLOSOne_formatting_sample_main_body.pdf>

**Response**：Thanks for your suggestion. We have carefully modified the manuscript according to the standard template to ensure that the manuscript meet the style requirements.

2. Please note that PLOS ONE has specific guidelines on code sharing for submissions in which author-generated code underpins the findings in the manuscript. In these cases, we expect all author-generated code to be made available without restrictions upon publication of the work. Please review our guidelines at https://journals.plos.org/plosone/s/materials-and-software-sharing#loc-sharing-code and ensure that your code is shared in a way that follows best practice and facilitates reproducibility and reuse.

**Response**: Thanks for your comments. The models employed in this article combined utilization of several platforms. Currently, the programs codes for each section are still in a disorganized form and are quite chaotic. We are also currently trying to develop and optimize this model, thus we are inconvenient to upload the code at this moment. If you have any future requests, please contact us again.

3. Thank you for stating the following financial disclosure: [The research is supported by the National Natural Science Foundation of China（51875090）].

Please state what role the funders took in the study.  If the funders had no role, please state: "The funders had no role in study design, data collection and analysis, decision to publish, or preparation of the manuscript."

If this statement is not correct you must amend it as needed.

Please include this amended Role of Funder statement in your cover letter; we will change the online submission form on your behalf.

**Response**: Thanks for your reminder. The funders played roles in decision to publish and preparation of the manuscript. The approach discussed in this paper is one of the quality control methods employed in this funder. The technique intends to offer algorithmic assistance for defect detection and quality prediction of rolling bearing components in CNC machine tools, setting the groundwork for ongoing research in the area of intelligent manufacturing.

4. Thank you for uploading your study's underlying data set. Unfortunately, the repository you have noted in your Data Availability statement does not qualify as an acceptable data repository according to PLOS's standards.
At this time, please upload the minimal data set necessary to replicate your study's findings to a stable, public repository (such as figshare or Dryad) and provide us with the relevant URLs, DOIs, or accession numbers that may be used to access these data. For a list of recommended repositories and additional information on PLOS standards for data deposition.

**Response**: Thanks for your kind suggestion. we have updated the research data in the public repository Kaggle which is in the list of recommended repositories and additional information on PLOS standards for data deposition., the relevant URLs are as follows which are also be provided in online submission;

<https://www.kaggle.com/datasets/dzt1503/cwru-bearing-dataset12k>

Additional Editor Comments:

1) Please highlight the innovations and novelties of the paper.

**Response**: Thanks for your useful suggestion. We have added some better and more conspicuous descriptions of the innovations and novelties of the paper, notably in the section of introduction.

2) Please indicate the motivation of the research and the methodologies' combination. Why these methods can be combined and what is the benefit for this type of combination

**Response**: Thanks for your suggestion. We have presented a more extensive and theoretical explanation of the combinations in DBO-VMD based on mathematical concepts, while the IWCCSCA-SDAE part describes the optimization capabilities of IWCCSCA and supports it with experiments.

3) The main challenge of bearing fault diagnosis has not been given clearly.

**Response**: Thanks for your valuable suggestion. We have added brief and clear descriptions of the main challenge of bearing fault diagnosis for feature extraction and diagnostic models, respectively.

4) Some standard method such as VMD is well-established, the explanation can be simplified to shorten the paper.

**Response**: Thanks for your reminder. The explanation of standard methods such as VMD, SCA, SDAE, etc. which have been used in the paper has been simplified.

5) Nearly all the formula needs to be updated including all the symbols in the equations should be pre-defined clearly before usage.

**Response**: Thanks for your kind reminder. We have updated the formula and explained the symbols before usage, assure that the symbols in the equations are pre-defined.

6) A pseudocode needs to be given to show the implementation flow of the proposed method.

**Response**: Thanks for your valuable suggestion. A pseudocode has been supplied in Table 1, which depicts the implementation flow of the building of feature vector.

7) Please explain the source of the experimental data, is the dataset a benchmark?

**Response**: Thanks for your valuable comments. The experimental data is the dataset of CWRU, which is a benchmark in the field of bearing fault diagnosis and an open dataset, and we have made a statement of the dataset in the paper.

8) A proof reading is highly recommended as the current typos and gramma problem affect the readability of the paper.

**Response**: Thanks for your suggestion. We have proofread the paper and corrected the typos and grammar problem that existed. We have also refined the English presentation to increase the readability of the paper. (Due to the enormous amount of correction and editing, this portion is not marked in the edited version)

Comment for the Reviewer#1:

1. Innovative expressions should be clear to avoid merely piling up methods. The paper combines various algorithms, but lacks sufficient justification for why these methods were chosen. The need for each method and how they work together to address the core challenge of rolling bearing fault diagnosis needs to be clearly stated.

**Response**: Thanks for your valuable suggestion. We have added brief and clear descriptions of the main challenge of bearing fault diagnosis for feature extraction and diagnostic models, respectively. And it can explain to some extent why these methods were chosen.

2.Tanimoto coefficients are commonly used for set similarity comparison, whereas permutation entropy and kurtosis reflect signal complexity and shock characteristics. However, the physical meaning and mathematical compatibility of combining these three as fitness functions have not been fully explained. It is recommended to verify the contribution of each index within the fitness function through an ablation experiment or to provide a theoretical basis. ok

**Response**: Thanks for your kind reminder. We presented a deeper theoretical and mathematical explanation of these three coefficients, offering an explanation of the reasons for their combination. By explaining the meaning of these parameters and their role in fault signals, we finally derived the formula.

3.Hyperparameter sensitivity experiments need to be supplemented to evaluate the effectiveness of the improvement.

**Response**: Thanks for your valuable comments. This is a productive and forward-looking question. This paper focuses on choosing relevant hyperparameters using optimal approaches to develop more successful models, but does not concentrate on the particular link between hyperparameters and models. As demonstrated in the experimental portion of this research, the hyperparameters picked by different optimization techniques do have an influence on the experimental outcomes. In further study, we will concentrate on examining the particular influence of hyperparameters on model performance.

4.There is insufficient experimental comparison. The current mainstream methods, such as deep convolutional networks, are not clearly compared. figure

**Response**: Thanks for your suggestion. We have added a model utilizing CNN for convolution optimization and feature extraction, and then using SVM for classification, as a comparison model in the experimental part to demonstrate the superiority of this technique.

5.Computational costs are not discussed. The method involves multiple optimization and feature extraction, which may have high computational complexity. The time cost needs to be analyzed compared to a lightweight model. ok

**Response**: Thanks for your valuable comment. We added a set of experiments based on model iterations and time consumption to verify that our method proposed in this paper has no evident drawbacks in terms of time computation.
